# Hyperbaric Oxygen Enabled a Transition to Oral Steroids in an Acute Severe Ulcerative Colitis Flare

**DOI:** 10.1093/crocol/otae017

**Published:** 2024-03-22

**Authors:** Megan M Hennessey, Sara R Zelman, Pam M Hannigan, Kimberly B Wilkinson, Corey A Siegel, Jay C Buckey

**Affiliations:** Department of Internal Medicine, Dartmouth-Hitchcock Medical Center, Lebanon, NH, USA; Division of Gastroenterology and Hepatology, Department of Internal Medicine, Dartmouth-Hitchcock Medical Center, Lebanon, NH, USA; Dartmouth-Hitchcock Medical Center, Lebanon, NH, USA; Dartmouth-Hitchcock Medical Center, Lebanon, NH, USA; Section Chief of Gastroenterology and Hepatology, Co-Director of the Inflammatory Bowel Disease (IBD) Center at Dartmouth-Hitchcock Medical Center, Lebanon, NH, USA; Geisel School of Medicine at Dartmouth, Hanover, NH 03755, USA

**Keywords:** inflammatory bowel disease, ulcerative colitis, acute severe ulcerative colitis, hyperbaric oxygen therapy, steroid-refractory disease

## Abstract

**Background:**

Ulcerative colitis (UC) is characterized in part by a dysregulated response to tissue hypoxia. While intravenous (IV) steroids are the mainstay of treatment for acute severe UC (ASUC), up to one-third of patients are refractory to steroids alone and require rescue therapy.

**Case Description:**

A 71-year-old female with extensive UC on infliximab presented with abdominal pain and more than 10 bloody bowel movements per day. Her infliximab concentration was undetectable with a positive antibody level. Flexible sigmoidoscopy on hospital day (HD)1 showed Mayo 3 colitis; biopsies for CMV were negative. She was started on hydrocortisone IV with improvement in her CRP from 56 to 40 mg/L. She also received 1 dose of vedolizumab. Hyperbaric treatments were offered but declined. By HD5, she was clinically improved, with a CRP of 9 mg/L. She was transitioned from IV to oral steroids. After starting oral steroids her symptoms relapsed, her CRP increased from 9 to 48 mg/L, and IV steroids were reinitiated on HD6. Hyperbaric medicine was reconsulted and she completed 5 hyperbaric oxygen (HBO2) treatments (HD 7–11) with prompt reduction in CRP, stool frequency, and bleeding. After 3 HBO2 treatments, she transitioned successfully from IV to oral steroids on HD9.

**Conclusions:**

This case demonstrates the potential of HBO2 therapy to help UC patients transition successfully from IV to oral steroids who were previously refractory to de-escalation. HBO2 therapy may be considered as an adjunctive treatment for patients with ASUC to potentiate the effects of standard therapies and avoid progression to colectomy.

## Background

Ulcerative colitis (UC) is a chronic inflammatory bowel disease characterized in part by a dysregulated response to tissue hypoxia that leads to altered mucosal barrier function and activation of pathogenic T cells. This causes mucosal damage from cytokine release and hypoxia that can manifest clinically as disease flares with increased bowel movement frequency, bleeding, urgency, and abdominal pain.^1^

Currently, intravenous corticosteroids are the mainstay of treatment for acute severe UC (ASUC) flares. Unfortunately, approximately one-third of patients with ASUC are refractory to corticosteroid treatment alone and require rescue therapy during their hospitalization with small molecule or biologic agents to avoid urgent colectomy.^2–4^

Emerging evidence from a small controlled trial shows that hyperbaric oxygen therapy (HBO2) can be an effective short-term treatment option for patients hospitalized with moderate to severe UC flares.^1^ While HBO2 is often used adjunctively with IV steroids, there are case reports where HBO2 was successfully used as monotherapy to induce clinical remission.^3^ This may be secondary to local reduction of hypoxia in the bowel mucosa, decreases in proinflammatory cytokines, upregulation of hypoxia response pathways, and beneficial alterations to the host microbiome. HBO2 has been shown to reduce host neutrophil STAT3 and azurophilic granule activity and lead to changes in microbial composition that improve colitis activity.^5^

The beneficial clinical effects of HBO2 can be difficult to distinguish from other treatments received during UC flares, as many patients receive IV steroids and biologic therapy while hospitalized. Here we present a patient admitted with ASUC who was unable to transition from IV to oral steroids due to recurrent symptoms. HBO2 treatments were then added to her regimen. She received 5 HBO2 treatments, transitioned successfully to oral steroids, and left the hospital.

## Case Presentation

A 71-year-old female with extensive UC on infliximab presented with abdominal pain and more than 10 daily bloody bowel movements. She was initially diagnosed with UC 3 years prior; however, she declined treatment at that time due to concern about medication side effects. She was admitted 4 months ago with extensive pancolitis, and during that admission, she was started on infliximab and discharged on prednisone 40 mg with a planned taper. Prednisone was tapered down by 10 mg every 2 weeks until 20 mg, then tapered by 5 mg every 2 weeks until completion. She had received 4 infusions of infliximab as an outpatient. At the time of the current presentation, she had completed her last infusion 11 days prior and had been off prednisone for 1 month.

On admission, she was tachycardic but otherwise hemodynamically stable. Labs were notable for: White blood count 12 × 10(3) mcL, hemoglobin 11 g/dL, albumin 3.7 g/dl, and C-reactive protein (CRP) 56 mg/L. Her infliximab level was undetectable with an antibody level of 86.8 U/mL (upper limit of normal < 50 U/mL*).* Infectious studies including stool culture, Shiga toxin, Campylobacter, and C difficile were negative. Abdominal X-ray performed did not show toxic megacolon or signs of perforation. She was started on hydrocortisone 100 mg IV every 8 hours with improvement in her CRP from 56 to 40 mg/L. Rescue therapy options including accelerated infliximab, vedolizumab, and JAK inhibitors were considered. Accelerated infliximab was not selected given the undetectable infliximab level and high antibody level, which seemed unlikely to be overcome. JAK inhibitors were contraindicated due to a history of prior silent myocardial infarction and venous thromboembolism. Vedolizumab was chosen for a more favorable safety profile and was administered on hospital day (HD) 1. Flexible sigmoidoscopy on HD1 showed Mayo 3 colitis with negative biopsies for cytomegalovirus (CMV). Hyperbaric medicine was consulted; however, the patient declined this therapy.

On HD5, CRP downtrended to 9 mg/L and she was clinically improving with fewer bowel movements and minimal hematochezia, so she was transitioned from IV to oral steroids (prednisone 40 mg daily).

Unfortunately, after switching to oral steroids she developed increased abdominal pain, stool frequency, and blood in her stools. Her CRP increased rapidly from 9 to 48 mg/L, prompting re-initiation of IV steroids on HD6. Given her inability to tolerate switching from IV to oral steroids and concern that her UC flare may be medically refractory; colorectal surgery was consulted. Given the reliance on IV steroids and concern regarding the need for colectomy, hyperbaric medicine was reengaged.

**Figure 1.**
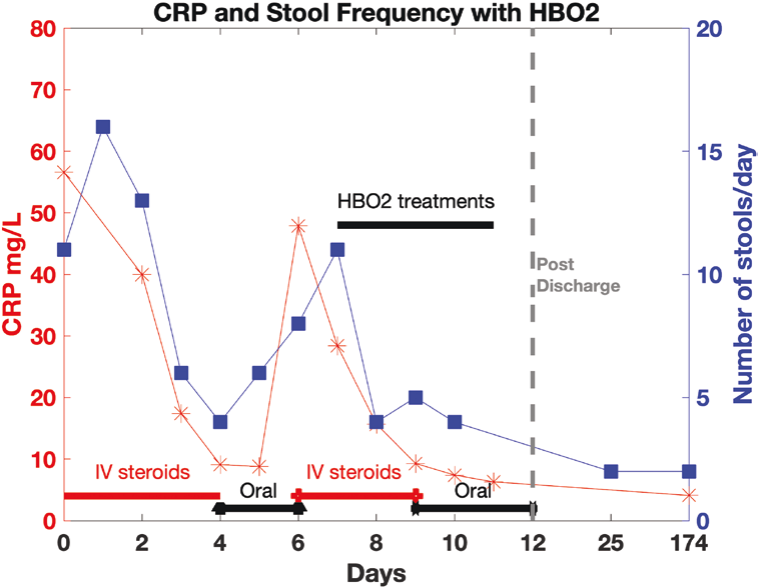
Red lines show C-reactive protein (CRP) levels. Blue lines show stool frequency. She was discharged on hospital day 12 and had a follow up appointment on day 25 after admission. Her most recent follow up appointment was 174 days post admission.

She completed 5, 90-minute hyperbaric treatments at 2.0 ATA (HD 7–11) with significant and prompt reduction in both CRP and stool frequency (Figure 1). CRP decreased from 28 to 6.3 mg/L. Stool frequency improved from 7 to 8/day to 4/day and bleeding stopped. After 3 hyperbaric treatments, she had significant clinical improvement with a downtrending CRP and was successfully transitioned from IV to oral steroids (prednisone 60 mg daily) on HD9. She was discharged on oral prednisone (60 mg daily with prolonged taper) with close outpatient follow-up and outpatient vedolizumab infusions. Follow-up post discharge showed sustained improvement; at 5 months she remained clinically in remission off steroids with 1–2 non-bloody bowel movements per day and a CRP of 4.1 mg/L.

## Discussion/Implications

Potential benefits of HBO2 include reducing the need for rescue therapies and preventing progression to colectomy. This was demonstrated in a phase 2A multi-center, double-blind, sham-controlled, randomized trial which showed that HBOT in addition to IV steroids for UC patients hospitalized for acute flares resulted in a significantly higher rate of clinical response and remission, and a lower rate of progression to infliximab or colectomy during hospitalization than patients who received steroids alone.^1^ A phase IIB HBO2 dose-finding trial was able to determine that 5 days of HBO2 was superior to 3 days when used as an adjunct with IV steroids with resolution or near complete resolution of rectal bleeding achieved in over 70% of patients after 5 days HBO2.^6^

In patients receiving HBO2 for UC flares, determining effectiveness is often difficult as patients almost always receive concomitant treatments. This case is unique as our patient initially declined HBO2 therapy, received IV steroids and failed the transition to oral steroids. She then initiated HBO2 therapy, transitioned successfully to oral steroids and left the hospital. This outcome is encouraging as it demonstrates the potential for HBO2 therapy to help patients transition successfully from IV to PO steroids who previously were refractory to de-escalation. This may be due to HBO2 potentiating the efficacy of corticosteroids or to a complementary ability to induce clinical remission by relieving hypoxia. The benefits of HBO2 likely extend beyond relieving tissue hypoxia. HBO2 has been also shown to reduce proinflammatory cytokines, alter the intestinal microbiome, and reduce both STAT3 activity and neutrophil degranulation.^5^

Since UC biologic medications can have a slow onset of response, HBO2 can also help to bridge the gap between induction therapy and maintenance. This may help to reach clinical response faster and allow patients to initiate biologic therapy as outpatients.

This patient received vedolizumab on HD1 raising the possibility that her improvement was due to this drug rather than HBO2. The consensus in the literature, however, is that the onset of vedolizumab’s effects does not occur for 4–8 weeks after administration, with majority of patients in the VARSITY trial achieving clinical response by week 14.^7,8^ Therefore, for this patient, the induction of clinical remission was likely relying on the response to corticosteroids and HBO2 therapy. Nevertheless, the possibility exists that either vedolizumab or the combination of vedolizumab and HBO2 helped with the transition to oral medications in this case.

Overall, HBO2 therapy may be considered as an adjunctive treatment option for patients with severe UC flares, both to help potentiate effects of current therapies, avoid progression to colectomy, and expedite time to discharge. Future controlled studies investigating the potential benefits of HBOT in patients hospitalized with ASUC flares are warranted.

## Data Availability

No new data was created or analyzed.

## References

[1] Dulai PS , Buckey JC, Jr, Raffals LE, et al. Hyperbaric oxygen therapy is well tolerated and effective for ulcerative colitis patients hospitalized for moderate-severe flares: a phase 2a pilot multi-center, randomized, double-blind, sham-controlled trial. Am J Gastroenterol. 2018;113(10):1516-1523. 29453383 10.1038/s41395-018-0005-z

[2] Turner D , Walsh CM, Steinhart AH, Griffiths AM. Response to corticosteroids in severe ulcerative colitis: a systematic review of the literature and a meta-regression. Clin Gastroenterol Hepatol. 2007;5(3):103-110. 17142106 10.1016/j.cgh.2006.09.033

[3] Harlan NP , Roberts J, Siegel C, Buckey JC. Hyperbaric oxygen as successful monotherapy for a severe ulcerative colitis flare. Inflamm Bowel Dis. 2022;28(9):1474-1475. 35771656 10.1093/ibd/izac141

[4] Laharie D , Bourreille A, Branche J, et al.; Groupe d'Etudes Thérapeutiques des Affections Inflammatoires Digestives. Ciclosporin versus infliximab in patients with severe ulcerative colitis refractory to intravenous steroids: a parallel, open-label randomised controlled trial. Lancet. 2012;380(12):1909-1915. 23063316 10.1016/S0140-6736(12)61084-8

[5] Gonzalez CG , Mills RH, Kordahi MC, et al. The host-microbiome response to hyperbaric oxygen therapy in ulcerative colitis patients. Cell Mol Gastroenterol Hepatol. 2022;14(1). 10.1016/j.jcmgh.2022.03.008 PMC9117812 35378331

[6] Dulai PS , Raffals LE, Hudesman D, et al. A phase 2b randomised trial of hyperbaric oxygen therapy for ulcerative colitis patients hospitalised for moderate to severe flares. Aliment Pharmacol Ther. 2020;52(6):955-963. 32745306 10.1111/apt.15984

[7] Sands BE , Peyrin-Biroulet L, Loftus EV, Jr, et al.; VARSITY Study Group. Vedolizumab versus adalimumab for moderate-to-severe ulcerative colitis. N Engl J Med. 2019;381(1):1215-1226. 31553834 10.1056/NEJMoa1905725

[8] Sablich R , Urbano MT, Scarpa M, Scognamiglio F, Paviotti A, Savarino E. Vedolizumab is superior to infliximab in biologic naive patients with ulcerative colitis. Sci Rep. 2023;13(1):1816. 36725872 10.1038/s41598-023-28907-3 PMC9892496

